# Soil pH amendment alters the abundance, diversity, and composition of microbial communities in two contrasting agricultural soils

**DOI:** 10.1128/spectrum.04165-23

**Published:** 2024-06-25

**Authors:** Ruonan Xiong, Xinhua He, Nan Gao, Qing Li, Zijian Qiu, Yixin Hou, Weishou Shen

**Affiliations:** 1 Jiangsu Key Laboratory of Atmospheric Environment Monitoring and Pollution Control, Collaborative Innovation Center of Atmospheric Environment and Equipment Technology, School of Environmental Science and Engineering, Nanjing University of Information Science and Technology, Nanjing, China; 2 School of Biological Sciences, University of Western Australia, Perth, Western Australia, Australia; 3 Department of Land, Air and Water Resources, University of California at Davis, Davis, California, USA; 4 National Engineering Research Center for Biotechnology, School of Biotechnology and Pharmaceutical Engineering, Nanjing Tech University, Nanjing, China; 5 Institute of Soil Health and Climate-Smart Agriculture, Nanjing University of Information Science and Technology, Nanjing, China; USDA-ARS-NPRL, Dawson, Georgia, USA

**Keywords:** soil pH, agricultural soil, bacterial community, fungal community, diversity, composition

## Abstract

Soil microorganisms are the most active participants in terrestrial ecosystems, and have key roles in biogeochemical cycles and ecosystem functions. Despite the extensive research on soil pH as a key predictor of microbial community and composition, a limitation of these studies lies in determining whether bacterial and/or fungal communities are directly or indirectly influenced by pH. We conducted a controlled laboratory experiment to investigate the effects of soil pH amendment (+/- 1–2 units) with six levels on soil microbial communities in two contrasting Chinese agricultural soils (pH 8.43 in Dezhou, located in the North China Plain, Shandong vs pH 6.17 in Wuxi, located in the Taihu Lake region, Jiangsu, east China). Results showed that the fungal diversity and composition were related to soil pH, but the effects were much lower than the effects of soil pH on bacterial community in two soils. The diversity and composition of bacterial communities were more closely associated with soil pH in Wuxi soils compared to Dezhou soils. The alpha diversity of bacterial communities peaked near *in situ* pH levels in both soils, displaying a quadratic fitting pattern. Redundancy analysis and variation partition analysis indicated that soil pH affected bacterial community and composition by directly imposing a physiological constraint on soil bacteria and indirectly altering soil characteristics (e.g., nutrient availability). The study also examined complete curves of taxa relative abundances at the phylum and family levels in response to soil pH, with most relationships conforming to a quadratic fitting pattern, indicating soil pH is a reliable predictor. Furthermore, soil pH amendment affected the transformation of nitrogen and the abundances of functional genes involved in the nitrogen cycle, and methane production and consumption. Overall, results from this study would enhance our comprehension of how soil microorganisms in contrasting farmlands will respond to soil pH changes, and would contribute to more effective soil management and conservation strategies.

**IMPORTANCE:**

This study delves into the impact of soil pH on microbial communities, investigating whether pH directly or indirectly influences bacterial and fungal communities. The research involved two contrasting soils subjected to a 1–2 pH unit amendment. Results indicate bacterial community composition was shaped by soil pH through physiological constraints and nutrient limitations. We found that most taxa relative abundances at the phylum and family levels responded to pH with a quadratic fitting pattern, indicating that soil pH is a reliable predictor. Additionally, soil pH was found to significantly influence the predicted abundance of functional genes involved in the nitrogen cycle as well as in methane production and consumption processes. These insights can contribute to develop more effective soil management and conservation strategies.

## INTRODUCTION

Soil microorganisms are diverse and abundant, known as Earth’s dark matter (1). It has been estimated that 1 g of soil contains thousands of microbial taxa, which raise challenges to investigate microbial diversity and community composition (2). Soil microorganisms have crucial roles in biogeochemical processes and in the maintenance of ecosystem functions in terrestrial ecosystems, such as driving nutrient cycling, determining crop productivity, regulating climate change (2–4), and helping determine ecosystem multifunctionality (5–7). Various factors, such as pH (8), inorganic nitrogen content (9), climate change (10), land-use change (11), and crop rotation (12), have been identified as drivers of microbial diversity and composition. Because bacteria and fungi are generally the dominant microorganisms in soil, it is necessary to understand their diversity, composition, and abundance in various ecosystems.

Over the past few decades, excessive application of chemical fertilizers and the massive emission of acid gases (sulfur dioxide and nitrogen oxide) have significantly resulted in soil acidification, particularly in agroecosystems (13, 14). Soil acidification has increased concentrations of soil H^+^ and Al^3+^, which results in suppressing plant and belowground communities (15). In order to increase soil pH, the application of high pH substances (e.g., liming) has been a common method, which also affects the soil microbial communities (16). On the other hand, soil salinization is also accelerated by increasing anthropogenic activities and climate change, such as land use intensification and the occurrence of more extreme climate events, particularly in arid and semi-arid areas (17, 18). Soil salinization denotes a land degradation process characterized by the accumulation of excessive soluble salts in soil, adversely affecting both plant growth and microbial communities (19, 20). Furthermore, the accumulation of sodium salts can indeed lead to a general increase in soil pH (19). It is well known that soil pH is vital in shaping microbial diversity and composition (21, 22). However, it remains poorly understood how pH affects soil microbial diversity, composition, and abundance in different ecosystems.

Variations in soil microbial communities have related with soil pH across different scales, including large continental scales (21, 23), land-use types at a given location (24), and small or sub-meter scales (25). For instance, the overall phylogenetic diversity and composition of bacterial communities were significantly correlated with soil pH at the continental scale (21). Soil pH was a key factor affecting the diversity, structure, interaction, and function of rhizosphere bacterial communities in acidic soil in artificial greenhouses (26). This phenomenon has also been observed in high elevation alkaline soils on the Tibetan Plateau (27). However, numerous soil and site characteristics (e.g., soil moisture, nutrient availability, and climate change) often co-vary with changes in soil pH. This presents a limitation as it becomes challenging to discern whether microbial communities are directly or indirectly influenced by soil pH. Further controlled experiments are necessary to elucidate whether alterations in soil pH alone can drive the observed patterns.

Soil pH has been found to be a reliable predictor of bacterial abundance at the phylum level by correlation analysis (22, 23, 26, 28, 29). These investigations have revealed varying relationships, characterized by either linear or quadratic associations, between the relative abundances of dominant bacterial phyla and soil pH. The phylum level provides a broad overview of ecological patterns and the general distribution of major microbial groups. However, it may overlook finer-scale differences present at lower taxonomic levels, such as family, genus, or species, which can be crucial for understanding specific functions and interactions within the community. The relationships between the fungal community and soil pH have garnered less attention compared to bacteria, primarily due to the relatively modest effect of soil pH on the fungal community (22). Moreover, most studies only focused on soil acidification; a complete response of soil microbial community to a range of soil pH has not been determined. Therefore, it is essential to explore the complete dynamics of diversity, composition, and abundance of bacterial and fungal communities as possible responses across a soil pH gradient in different soil types.

The changes in agricultural soil pH are mainly influenced by anthropogenic activities. The North China Plain and the Taihu Lake region are two of the most intensive double-cropping regions in China, which are also the hotspots of greenhouse gas emissions from agricultural sources (30). Due to land-use intensification, soil acidification is a major problem in the Taihu Lake region, whereas soil acidification and soil salinization coexist in the North China Plain (13, 18). These processes are closely related to soil pH, and the impacts of pH changes on soil microorganisms and their ecological functions are still unclear in these two regions. In the present study, we conducted a soil pH incubation experiment with six amendment pH levels in two contrasting agricultural soils from the North China Plain and the Taihu Lake regions, to investigate direct effects of pH changes on soil bacterial and fungal communities by high-throughput sequencing. Using two contrasting agricultural soils, the objectives of this study were to determine (i) the diversity and composition of soil bacterial and fungal communities across a pH gradient in a controlled experiment, (ii) whether the microbial communities are shaped directly or indirectly by soil pH, and (iii) the complete response curves of the dominant microbial phyla at the phylum and family levels to pH gradients. Here, we hypothesized that alpha diversity of the microbial communities would peak near *in situ* pH. We assumed that soil pH would influence microbial composition by physiology and nutrient limitation. Additionally, we anticipated that the majority of taxa abundances at phylum and family levels would respond to pH by quadratic fitting, and the patterns of change would differ between contrasting cropping systems.

## MATERIALS AND METHODS

### Site description

Soils were collected from a wheat-maize rotation field in the North China Plain in Dezhou (37°18′N, 116°29′E), Shandong, and from a rice-wheat rotation field in the Taihu region in Wuxi (31°18′N, 119°55′E), Jiangsu, China, in June 2021 and August 2021, respectively. The current agricultural practice involves irrigated winter wheat and summer maize rotations in Dezhou. Winter wheat is sown in early October and harvested in early June the following year, after which summer maize is sown at once and reaped at the end of September. The current agricultural practice in Wuxi involves waterlogged summer rice and upland winter wheat rotations. Rice is sown in early June and reaped at the end of October, after which wheat is immediately sown and reaped at the end of May the following year. The annual application rate of synthetic nitrogen reaches about 550–600 kg of nitrogen per hectare in the two intensive double-cropping systems. No herbicides but pesticides are applied to plants when required. The Dezhou region has a warm temperate monsoon climate, with an average annual temperature of 13.8°C and an average annual precipitation of 512 mm. The Wuxi region has a subtropical monsoon climate, with an average annual temperature of 15.7°C and an average annual precipitation of 1,177 mm. The two types of soil are classified as Ochric Aquic Cambosols and Stagnic Anthrosols according to the Chinese soil taxonomy. At each site, we adopt a systematic random sampling method by randomly selecting 10 sampling points within a 100 m^2^ (10 m by 10 m) grid. At each selected sampling point, soil samples were collected by an auger at 0- to 20-cm depth. These soil samples were then composited as one representative sample for each site. The composite samples were immediately kept at 4°C and later stored in the laboratory. The collected soil samples were air dried for 5–7 days after the removal of debris, then sieved through 2-mm mesh and thoroughly homogenized. Soil samples were archived at 4°C for subsequent experiments 2 months later. The physicochemical properties of soils are shown in Table S1.

### Experimental design and soil sampling

The *in situ* pH values (measured in a 1:2.5 soil-to-water ratio) of soils collected from Dezhou and Wuxi were 8.43 and 6.17, respectively. According to the pilot trials for soil pH amendment, six pH treatments were amended by 0, 4, 24, 34 mL 2 mM H_2_SO_4_ (H) or 1.8, 4 mL 1 mM NaOH (Na) for the Dezhou (D) soil that were labeled as 0D (control; D, Dezhou), 4HD, 24HD, 34HD or 1.8NaD, 4NaD; whereas by 0, 0.4, 1, 2 mL 2 mM H_2_SO_4_ or 2, 4 mL 1 mM NaOH for the Wuxi (W) soil that were labeled as 0W (control), 0.4HW, 1HW, 2HW or 2NaW, 4NaW (Table 1). The acid or alkaline was diluted to the volume that reached to 100% of soil maximum water holding capacity, then thoroughly mixed with 200-g soil in 500-mL jam bottles. Amended soil pH (pH_A_) was measured (a 1:2.5 soil-to-water ratio) by destructive sampling after 1 week of stationary incubation at 26°C (Table 1). Then, a nutrient solution containing urea (0.12 g of N) was mixed with soil to reach 80% of the maximum water holding capacity (31). The application of urea aimed to simulate the effects of pH on nitrogen transformation. Soil was then incubated at 26°C under dark and maintained approximately 80% of the maximum water holding capacity by supplementing with sterilized dH_2_O for a total of 31 days until the concentration of greenhouse gas N_2_O approached the atmospheric background value. Destructive sampling was then taken on days 4, 13, 21, and 31. The experiment included destructive sampling for amended soil pH (pH_A_) measurement and four destructive samplings after adding urea. Each treatment had 15 replicate samples, and each destructive sampling had three replicate samples. One part of the soil samples was stored at 4°C to measure soil physicochemical properties in less than a week. Another part of the soil samples was stored at −80°C until DNA extraction.

**TABLE 1:** Soil pH amendment in two contrasting agricultural soils^*a*^

Reagent type	Dezhou			Wuxi			
	Reagent volume (mL)	Treatment	pH_A_ (H_2_O)	Reagent volume (mL)	Treatment	pH_A_ (H_2_O)	
2 mM H_2_SO_4_							
	34	34HD	6.76 (0.08)	2	2HW	4.11 (0.03)	
	24	24HD	7.23 (0.02)	1	1HW	5.01 (0.07)	
	4	4HD	7.74 (0.01)	0.4	0.4HW	5.44 (0.05)	
	0	0D	8.43 (0.03)	0	0W	6.17 (0.07)	
1 mM NaOH							
	1.8	1.8NaD	9.05 (0.01)	2	2NaW	7.24 (0.10)	
	4	4NaD	9.49 (0.04)	4	4NaW	7.87 (0.06)	

^*a*^ Values are means of three replicate samples with SE in parentheses. pH_A_ represents the amended soil pH value of each treatment through the addition of acidic or alkaline solution before the experiment began. The pH value was the negative logarithm transformation of the hydronium ion concentration.

### Measurement of soil physicochemical properties

Soil pH was measured in a 1:2.5 soil-to-water ratio by a pH meter (HI 2211, HANNA Instruments, Italy) (32). Soil NH_4_^+^-N and NO_3_^−^-N were extracted with 2 M KCl and measured using the indophenol-blue colorimetric and double wavelength methods by an ultraviolet-visible spectrophotometer (UV1800, China), respectively (33). Soil total nitrogen (TN) and total carbon (TC) were measured using an elemental analyzer (UNICUBE, Elementar, Germany). Dissolved organic carbon (DOC) was extracted by deionized water using a soil-to-water ratio of 1:5 and was shaken at 250 rpm for 1 h. After centrifugation and filtration, the suspension was measured using a TOC-L CPH analyzer (Shimadzu, Japan) (34). Soil physicochemical properties were measured over a four destructive sampling period at days 4, 13, 21, and 31 of the experiment, and their dynamic changes are shown in Fig. S2 and S3.

### DNA extraction and high-throughput sequencing

Soil DNA was extracted from ~0.5 g fresh soil using a HiPure Soil DNA Mini Kit (Magen, China) according to the manufacturer’s manual. The DNA concentration was measured by micro volume spectrophotometer (NanoReady, Life Real, China). The extracted DNA samples were stored at −80°C. Samples at the end of the 31 days of incubation were selected for high-throughput sequencing. The V3-V4 region of 16S rRNA gene, which includes sequences from both bacteria and some archaea, was amplified to obtain ~466 bp products using the primers 341F (5′-CCTACGGGNGGCWGCAG-3′) and 806R (5′-GGACTACHVGGGTATCTAAT-3′) (35, 36). To identify fungal communities, the forward primer ITS3_KYO2 (5′-GATGAAGAACGYAGYRAA-3′) and the reverse primer ITS4 (5′-TCCTCCGCTTATTGATATGC-3′) were used to amplify ~381 bp of ITS2 region (37). PCR reactions, each containing 50 ng of template DNA, were performed in triplicate for each soil DNA sample using a thermal cycler (ABI 9700, Thermo, USA). The PCR amplification protocol consisted of initial denaturation at 95°C for 5 minutes, followed by 30 cycles of denaturation at 95°C for 1 minute, annealing at 60°C for 1 minute, extension at 72°C for 1 minute, and a final extension at 72°C for 7 minutes. The triplicate PCR products were combined and extracted from a 2% agarose gel, then purified utilizing the AxyPrep DNA Gel Extraction Kit (Axygen Biosciences, Union City, CA, USA). Subsequently, sequencing libraries with sample index tags were produced using the TruSeq DNA PCR-Free Sample Preparation Kit (Illumina, USA) following the manufacturer’s recommendations. The quality of the library was evaluated using the Agilent Bioanalyzer 2100 system. Sequencing was performed on an Illumina NovaSeq 6000 sequencing system to obtain 2 × 250 bp paired-end reads at the Genedenovo Company, Guangzhou, China. The raw high-throughput data from this analysis were submitted to the National Center for Biotechnology Information (NCBI) Sequence Read Archive (SRA) under the accession number PRJNA970917.

### Bioinformatics analysis

Paired-end sequences were performed to trim, filter based on quality score, denoise, merge, and remove chimeras using DADA2 program (38) in QIIME2-2022.2 pipeline (39) to generate amplicon sequence variants (ASVs). A total of 1,476,085 high-quality sequences (ranging from 35,303 to 53,101 sequences per sample) and 50,399 ASVs were retained for bacterial 16S rRNA gene. A total of 3,512,809 high-quality sequences (ranging from 38,776 to 111,391 sequences per sample) and 4,985 ASVs were retained for fungal ITS gene. All ASVs were aligned with MAFFT and used to build a phylogenetic tree using FastTree via q2-alignment with default settings (40, 41). Rarefaction curves of bacterial 16S rRNA and fungal ITS genes indicated that the sequencing depths reached saturation (Fig. S1). Alpha diversity metrics and beta diversity metrics were estimated using the q2-diversity after rarefying samples of bacterial 16S rRNA and fungal ITS genes to 35,303 and 38,776 sequences per sample, respectively. Sequences of bacterial 16S rRNA and fungal ITS genes were taxonomically classified using classify-sklearn naive Bayes classifiers trained on the SILVA SSU database 138 (https://www.arb-silva.de/documentation/release-138/) and UNITE v.8.3 (https://unite.ut.ee/repository.php) via the q2-feature-classifier, respectively (42–44). The potential functional predictions based only on 16S rRNA gene sequences were conducted by PICRUSt2 (v2.5.0) (45).

### Statistical analyses

Spearman correlations among environmental factors in soils were analyzed at *P* < 0.05 using the psych and corrplot packages in the R v4.2.2. The alpha diversity based on site and treatment was calculated using the Shannon index, and significance was determined using the Kruskal–Wallis pairwise tests via q2-diversity in QIIME2 platform. The best-fit modeling of the Shannon index and environmental factors was performed by basicTrendline package in R, using linear and polynomial (quadratic) functions. To evaluate the dissimilarities in bacterial and fungal communities among treatments, principal coordinate analysis (PCoA) based on weighted UniFrac distance, analysis of similarities (ANOSIM), permutational multivariate analysis of variance (PERMANOVA), and permutational multivariate analysis of dispersion (PERMDISP) were performed using q2-diversity in QIIME2 platform. PERMDISP was completed together with PERMANOVA to evaluate the dispersion. The degree of collinearity of each environmental factor was measured using the variance inflation factor (VIF). The relationships between community composition and soil environmental factors (VIF < 10) were assessed based on weighted UniFrac distance using transformation-based redundancy analysis (tb-RDA) by a vegan package (v2.6-4) at the genus level. Individual effect of each soil environmental factor for total sites or single site was examined by the rdacca.hp package (46). To investigate the responses of bacterial and fungal communities’ relative abundances at the phylum and/or family level to amended soil pH, we conducted a best-fit modeling using linear and polynomial (quadratic) functions in R. In fact, the pH scale is logarithmic, defined as the decimal logarithm of the reciprocal of the hydrogen ion concentration. The pH values, being the negative logarithm transformation of the hydronium ion concentration, were appropriate for conducting regression analysis and parametric analysis, as the transformation met the assumptions required for such analyses. Analysis of composition of microbiomes (ANCOM) was used to identify features that differ in absolute abundance among treatments and sites at the phylum level in QIIME2 platform (47). For all ANCOM analyses, we screened for features with a minimum frequency of 50 that appear in ≤4 samples to remove noise caused by low abundance features. The predicted abundances of functional genes that participate in the nitrogen cycle, and methane production and consumption by PICRUSt2 were shown with a heatmap after Z-Score in R. Microbiome multivariable association with linear models (MaAsLin 2) was used to test differential relative abundances for functional genes by Maaslin2 package (48).

## RESULTS

### Response of soil physicochemical properties to pH amendment

**Fig 1:**
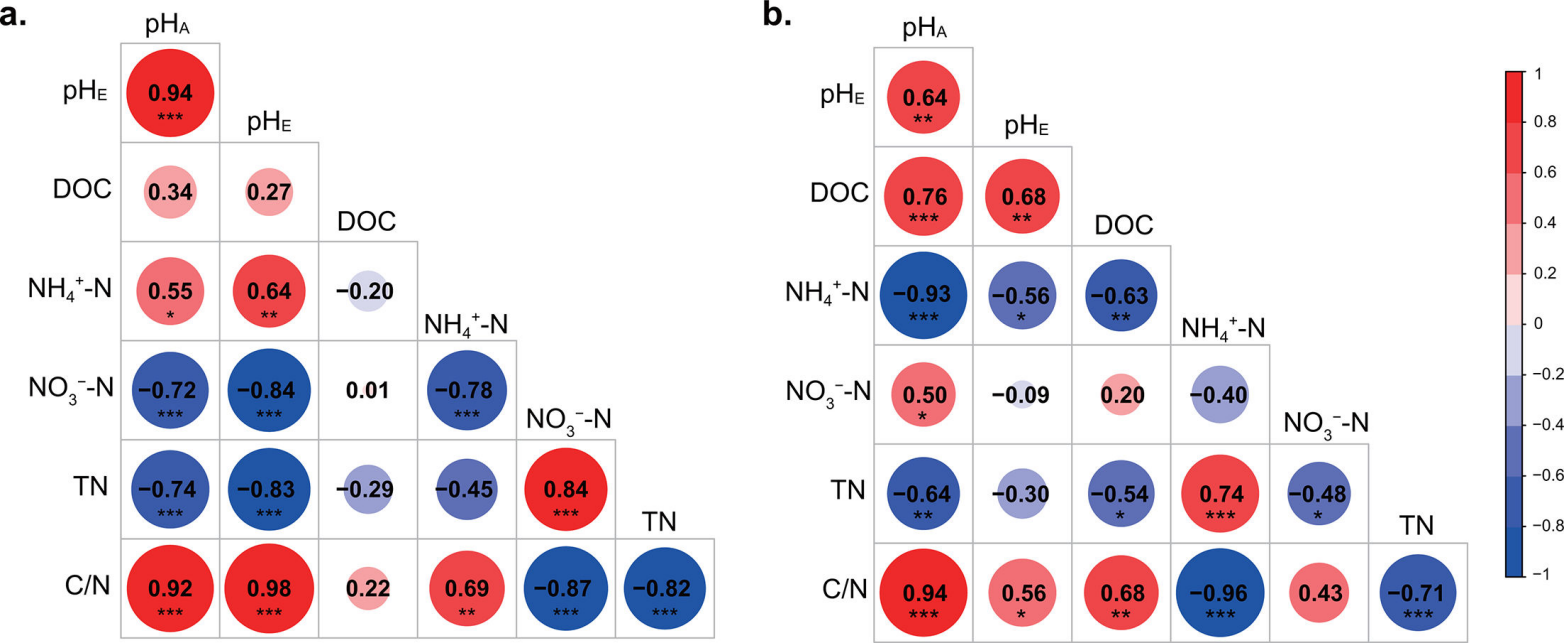
Spearman correlations between soil physicochemical properties at the end of the experiment and amended pH at the Dezhou (a) and Wuxi (b) sites. pH_A_ represents the amended soil pH value through the addition of acidic or alkaline solution before the start of the experiment, whereas pH_E_ represents the soil pH value at the end of the experiment. Color gradient and circle size denote Spearman’s correlation coefficients. The pH value was the negative logarithm transformation of the hydronium ion concentration. Asterisks denote different significance levels: *P < 0.05, **P < 0.01, and ***P < 0.001.

Amended soil pH (pH_A_) in the Dezhou agricultural soil resulted in similar gaps remaining in soil pH at the end of the experiment (Fig. S2), with a significant positive correlation with end-time pH (pH_E_) (Fig. 1a). 34HD and 24HD treatments accelerated the conversion rate of nitrogen, therefore contained lower NH_4_^+^-N content and higher NO_3_^−^-N content at the end of the experiment (Fig. S2). The pH_A_ was significantly positively correlated with NH_4_^+^-N and C/N content, while negatively correlated with NO_3_^−^-N and TN content (Fig. 1a).

The responses of soil physicochemical properties to pH amendment in Wuxi differed from those in Dezhou (Fig. 1b). The increase of pH and production of NH_4_^+^-N in 2HW treatment were at a sluggish pace (Fig. S3), possibly due to the hindered decomposition of urea. The pH_A_ was significantly positively correlated with the pH_E_, DOC, NO_3_^−^-N, and C/N, and significantly negatively correlated with NH_4_^+^-N and TN (Fig. 1b).

### Response of microbial diversity to pH amendment

**Fig 2:**
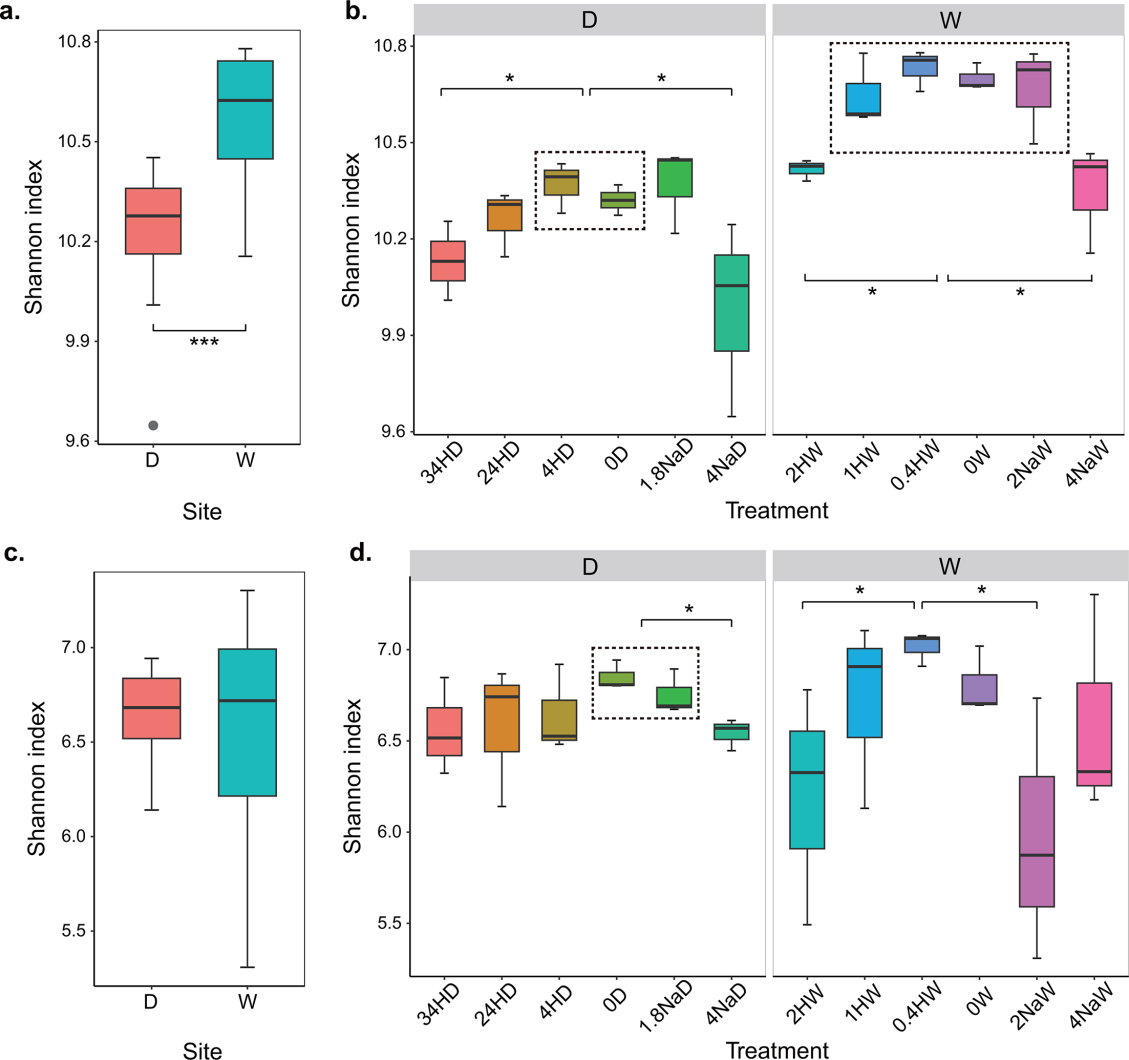
Soil bacterial and fungal alpha diversity affected by site and treatment. The Shannon indices were affected by site (*n* = 18) or by treatment (*n* = 3) for bacterial (a or b) and fungal (c or d) community, respectively. Significant differences were based on the Kruskal–Wallis pairwise test. The treatments in the dotted box are significantly different from the treatment of the connecting line. Boxes represent the interquartile range (IQR, 25%–75% of the data). The median values are represented by the bar inside each box, and whiskers extend to values within 1.5 times the IQR. Outliers, data points lying beyond this range, are depicted as gray points. Asterisks denote different significance levels: *P < 0.05, **P < 0.01, and ***P < 0.001. D, Dezhou; H, H_2_SO_4_; Na, NaOH; and W, Wuxi.

The Shannon index of the bacterial community in Wuxi was significantly higher than that in Dezhou (*P* < 0.001) (Fig. 2a). For the bacterial community, the relationships between the Shannon indices of bacterial community and the amended pH conformed to the quadratic fitting in Dezhou (R^2^ = 0.42, *P* < 0.05) and Wuxi (R^2^ = 0.68, *P* < 0.001) soils (Fig. S4a and b). Alpha diversity of the bacterial community was highest near the *in situ* pH (control) in both Dezhou and Wuxi soils. The Shannon indices of 34HD and 4NaD treatments were significantly lower than those of 0D control and 4HD treatment in Dezhou (*P* < 0.05) (Fig. 2b). The Shannon indices of 2HW and 4NaW treatments were significantly lower than those of other treatments and 0W control in Wuxi (*P* < 0.05). Except pH_A_, none of the soil physicochemical properties were significantly associated with the alpha diversity of bacterial community in both soils.

The alpha diversity of the fungal community was similar between Dezhou and Wuxi soils (Fig. 2c). None of the soil physicochemical properties including pH_A_ (Fig. S4c and d) were significantly associated with the alpha diversity of fungal community in both Dezhou and Wuxi soils. The Shannon index of 4NaD treatment was significantly lower than that of 0D control and 1.8NaD treatment in Dezhou (*P* < 0.05) (Fig. 2d). The Shannon indices of 2HW and 2NaW treatments were significantly lower than those of 0.4HW treatment in Wuxi (*P* < 0.05).

### Response of microbial composition to pH amendment and related key drivers

**Fig 3:**
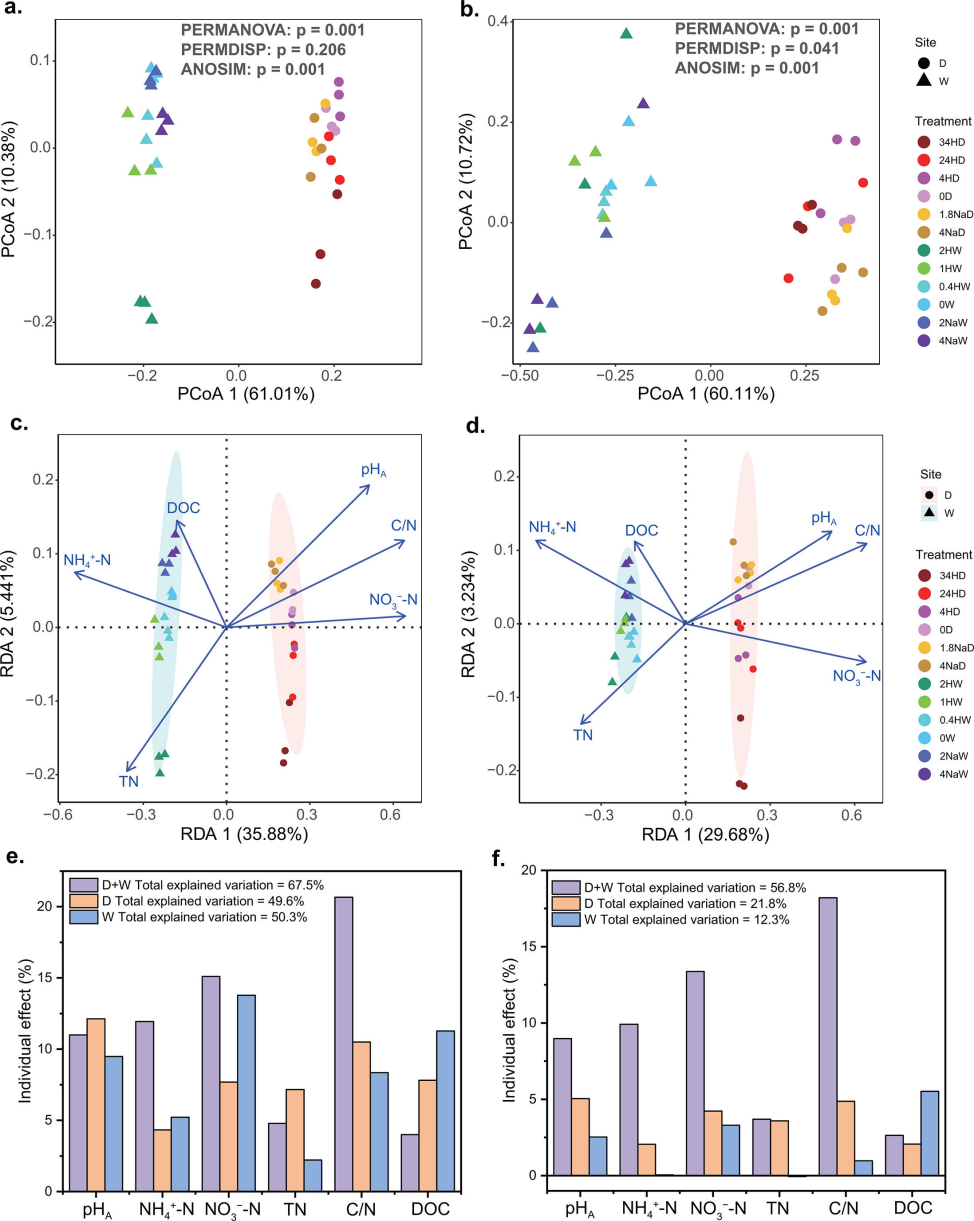
Variation in microbial communities induced by pH manipulation and the impacts of soil physicochemical properties on microbial communities. PCoA plot based on weighted UniFrac distance for bacterial (a) and fungal (b) communities at the ASV level. The statistical significance of PERMANOVA, PERMDISP, and ANOSIM across all treatments was assessed via 999 permutations test. Relationships between the community compositional structures and soil physicochemical properties for bacteria (c) and fungi (d) using tb-RDA based on weighted UniFrac distance at the genus level. The percentage in parentheses represents the variation explained by each axis. The confidence ellipse represents a 95% confidence level. Individual impact of each soil physicochemical property to bacterial (e) and fungal (f) communities in the two sites or single site. pH_A_ represents the amended soil pH value of each treatment through the addition of acidic or alkaline solution before the experiment began. The pH value was the negative logarithm transformation of the hydronium ion concentration. D, Dezhou; H, H_2_SO_4_; Na, NaOH; and W, Wuxi.

Principal coordinate analysis based on the weighted UniFrac distance showed that the bacterial community was clustered by site (Fig. 3a). The PERMDISP of bacterial community indicated no differences in dispersion among all treatments. The PERMANOVA and ANOSIM of bacterial community both denoted significant differences in soil bacterial community composition across all treatments, mainly due to differences in sites. However, their pairwise tests showed no significant differences in soil bacterial community composition among all treatments.

The RDA and variation partition analysis quantified the impact of each environmental factor on the variations in bacterial communities in two sites or single site at the genus level (Fig. 3c and e). Results from RDA showed the maximum degree of correlation between pH_A_ and bacterial community composition when only focused to the contribution of RDA 1 and RDA 2 axes to the variance (Fig. 3c). Individual effect of each soil environmental factor to the total explained variation was examined by rdacca.hp package (Fig. 3e). The variations in bacterial communities of Dezhou soil across treatments (0D, 4HD, 24HD, 34HD, 1.8NaD, and 4NaD) were mainly determined by pH_A_, C/N, DOC, and NO_3_^−^-N. The variations in bacterial communities of Wuxi soil across treatments (0W, 0.4HW, 1HW, 2HW, 2NaW, and 4NaW) were mainly determined by NO_3_^−^-N, DOC, pH_A_, and C/N. The variations in bacterial communities of both sites across all treatments were mainly determined by C/N, NO_3_^−^-N, NH_4_^+^-N, and pH_A_.

The principal coordinate analysis showed that the fungal community was also clustered by site (Fig. 3b). The PERMDISP of fungal community indicated differences in dispersion among all treatments, so the PERMANOVA might be unreliable. However, both the PERMANOVA and ANOSIM manifested significant differences in soil fungal community composition across all treatments, and their pairwise tests showed no significant differences in soil fungal community composition among all treatments.

Results from RDA showed the maximum degree of correlation between C/N and fungal community composition when only focused on the contribution of RDA 1 and RDA 2 axes to the variance (Fig. 3d). The variations in fungal communities of Dezhou soil across treatments (0D, 4HD, 24HD, 34HD, 1.8NaD, and 4NaD) were mainly determined by pH_A_, C/N, NO_3_^−^-N, and TN (Fig. 3f). The variations in fungal communities of Wuxi soil across treatments (0W, 0.4HW, 1HW, 2HW, 2NaW, and 4NaW) were mainly determined by DOC, NO_3_^−^-N, and pH_A_. The variations in bacterial communities of both soils across all treatments were mainly determined by C/N, NO_3_^−^-N, NH_4_^+^-N, and pH_A_. The totally explained variation of environmental factors to fungal community was lower than that of bacterial community.

### Response of taxon abundance to pH amendment

**Fig 4:**
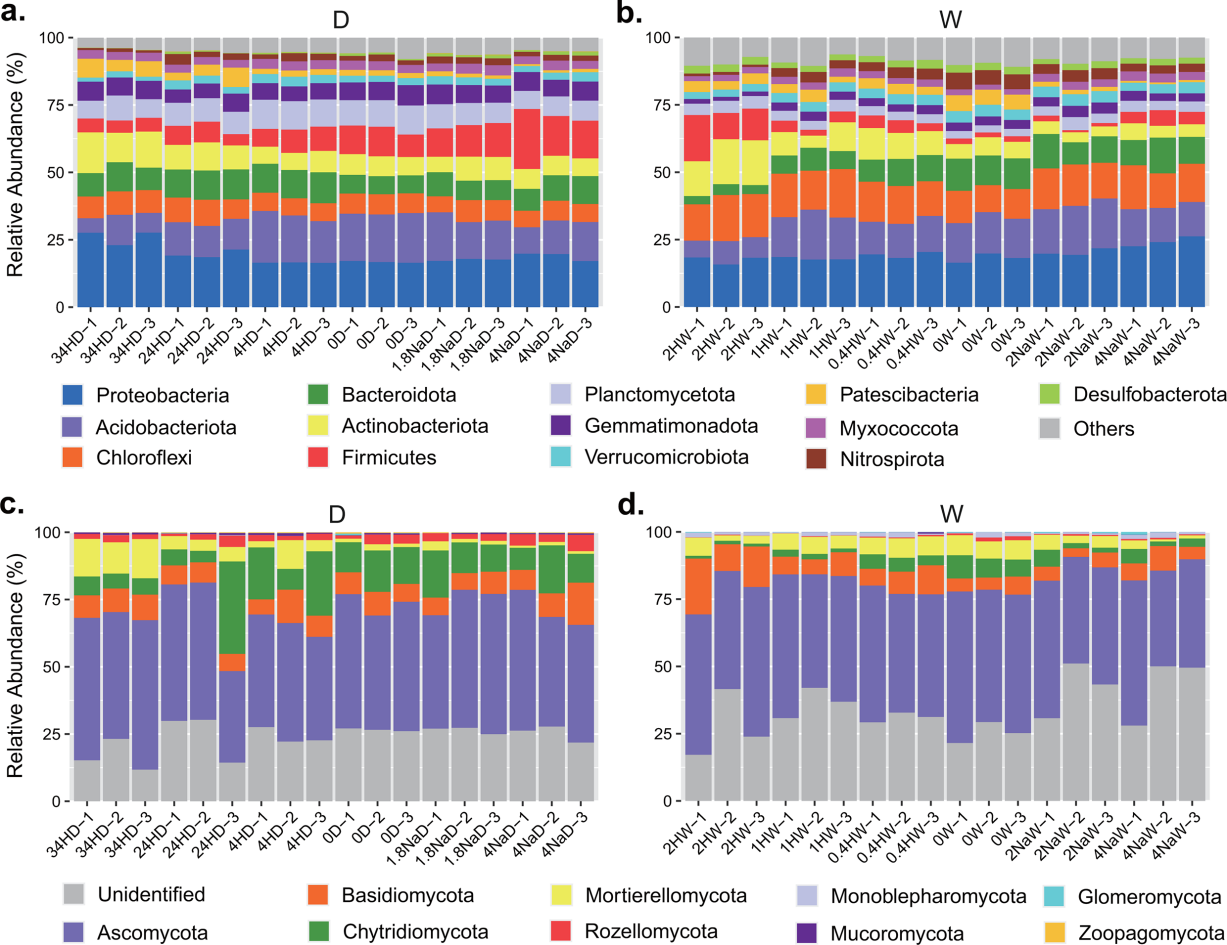
Relative abundances of the dominant bacterial (**a and b**) and fungal (**c and d**) phyla in two agricultural soils. Thirteen and nine of the most abundant taxa are displayed for bacterial and fungal communities, respectively. D, Dezhou; H, H_2_SO_4_; Na, NaOH; and W, Wuxi.

At the phylum level, the bacterial community in Dezhou soil primarily comprised Proteobacteria (19.3%), Acidobacteriota (13.8%), Firmicutes (9.6%), Bacteroidota (9.2%), Planctomycetota (8.7%), Actinobacteriota (8.6%), and Chlorolexi (7.5%) (Fig. 4a). Only the abundance of Patescibacteria was significantly different among treatments in Dezhou soil (Fig. S5a). Whereas, the bacterial community in Wuxi soil mainly consisted of Proteobacteria (19.6%), Chlorolexi (14.3%), Acidobacteriota (13.7%), Bacteroidota (8.8%), and Actinobacteriota (8.1%) (Fig. 4b). With the exception of Proteobacteria and Verrucomicrobiota, the abundances of other dominant bacterial phyla had significant differences among treatments in Wuxi soil (Fig. S5b). There were significant differences when comparing the abundances of dominant bacterial phyla between these two sites, except Proteobacteria, Actinobacteriota, Verrucomicrobiota, Patescibacteria, and Nitrospirota (Fig. S5c). Significantly greater abundances of Planctomycetota, Firmicutes, Gemmatimonadota, Myxococcota, Bacteroidota, and Acidobacteriota, but less abundances of Chloroflexi and Desulfobacterota were in the Dezhou soil than in the Wuxi soil.

The fungal community in Dezhou soil primarily comprised Ascomycota (46.6%), Chytridiomycota (12.8%), and Basidiomycota (8.3%) at the phylum level (Fig. 4c). Only the abundance of Mortierellomycota was significantly different among treatments in Dezhou soil (Fig. S6a). Whereas, the fungal community in Wuxi soil mainly consisted of Ascomycota (47.6%), Basidiomycota (7.9%), and Mortierellomycota (4.9%) (Fig. 4d). The abundances of Rozellomycota, Chytridiomycota, Mortierellomycota, Basidiomycota, and Ascomycota had significant differences among treatments in Wuxi soil (Fig. S6b). Significantly greater abundance of Monoblepharomycota, but less abundances of Zoopagomycota, Chytridiomycota, Rozellomycota, and Mucoromycota were observed in the Wuxi soil than in the Dezhou soil.

**Fig 5:**
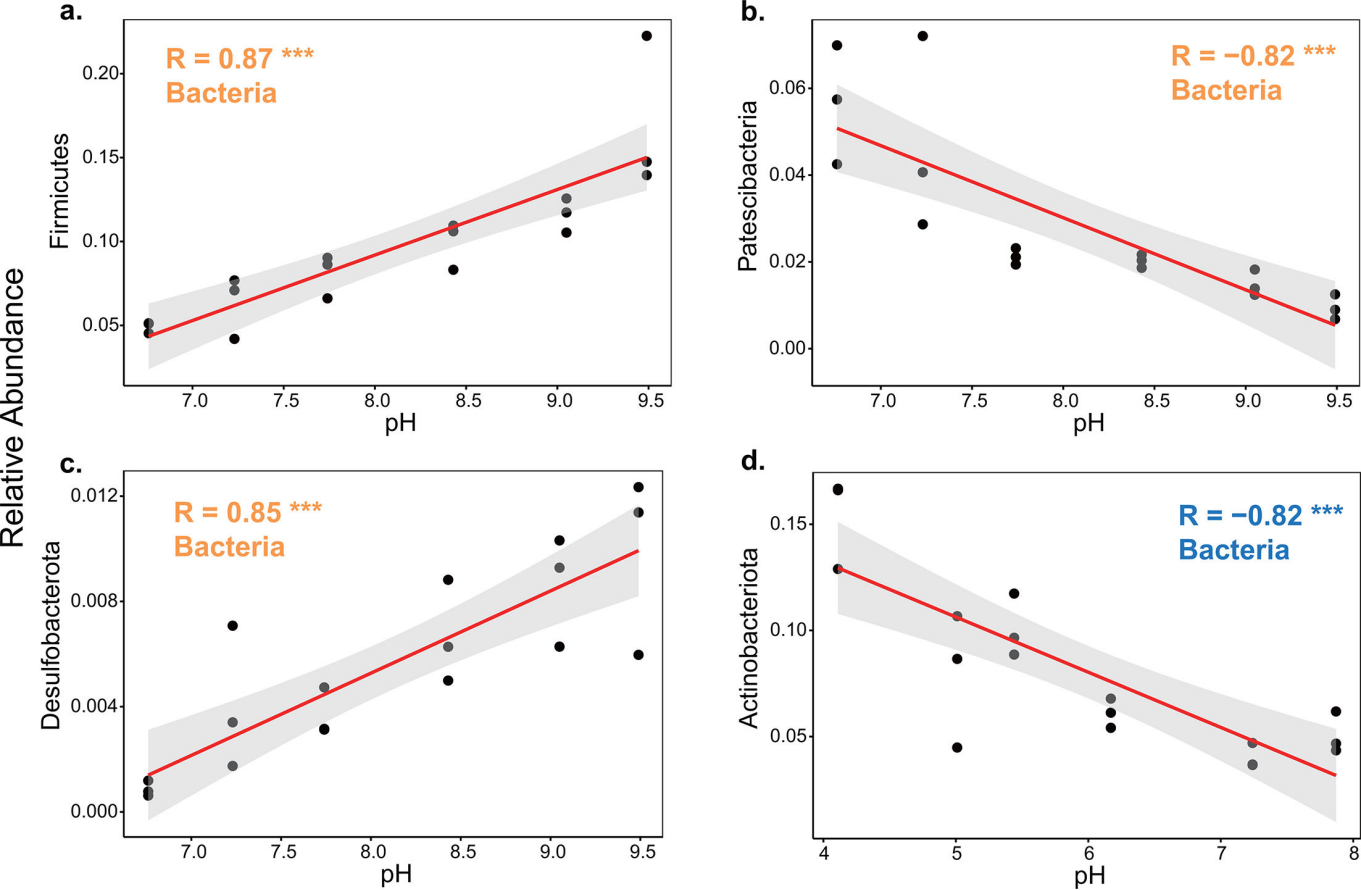
Correlations between the relative abundances of dominant bacterial phyla and amended soil pH at the Dezhou (**a, b, and c**) and Wuxi (d) sites. Lines represent the best-fit linear models to the data. Pearson correlations coefficients (**R**) are shown for each taxon with *P* values in yellow and blue at the Dezhou and Wuxi sites, respectively. Shadow represents a 95% confidence level. The pH value was the negative logarithm transformation of the hydronium ion concentration. Asterisks denote different significance levels: *P < 0.05, **P < 0.01, and ***P < 0.001.

**Fig 6:**
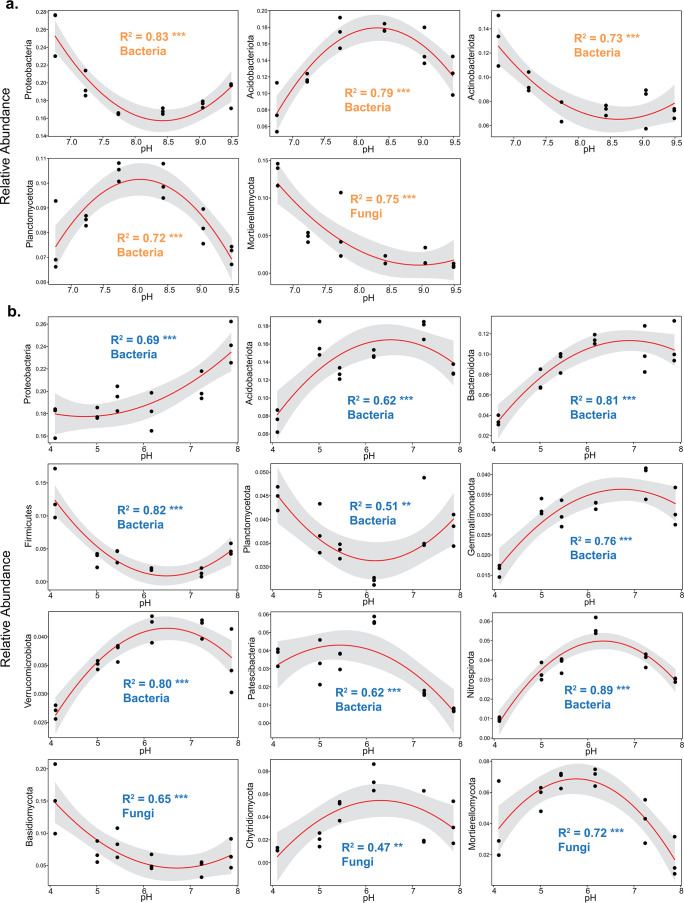
Relative abundances of dominant bacterial and fungal phyla in relation to amended soil pH at the Dezhou (a) and Wuxi (b) sites. Lines represent the best-fit quadratic models to the data. The coefficients of determination (**R^2^**) are shown for each taxon with *P* values. Shadow represents a 95% confidence level. The pH value was the negative logarithm transformation of the hydronium ion concentration. Asterisks denote different significance levels: *P < 0.05, **P < 0.01, and ***P < 0.001.

The dominant bacterial and fungal phyla responded differentially to changes in soil pH. The relationships between the relative abundances of dominant bacterial phyla and the amended pH conformed to a linear or quadratic fitting (Fig. 5 and 6). In Dezhou soil, the relative abundances of Firmicutes, Patescibacteria, and Desulfobacterota showed highly linear relationships with the amended pH (|R| > 0.8, *P* < 0.001) (Fig. 5a through c). Furthermore, the relative abundances of Firmicutes and Desulfobacterota increased while that of Patescibacteria decreased linearly with an increase of soil pH. Four bacterial phyla and one fungal phylum in relation to the amended pH conformed to the quadratic fitting in Dezhou soil (Fig. 6a). The relative abundances of Proteobacteria and Actinobacteriota were low near the *in situ* pH (control) and increased with the pH amendment, whereas the variations in Acidobacteriota and Planctomycetota showed the opposite trend. The relative abundance of Mortierellomycota decreased in a quadratic manner as soil pH increased. In Wuxi soil, the relative abundance of Actinobacteriota showed a negative linear correlation with the amended pH (R = −0.82, *P* < 0.001) (Fig. 5d). Nine bacterial phyla and three fungal phyla in relation to the amended pH conformed to the quadratic fitting in Wuxi soil (Fig. 6b). The relative abundances of Firmicutes, Planctomycetota, and Basidiomycota were least near the *in situ* pH and increased with the pH amendment. However, the relative abundances of Acidobacteriota, Bacteroidota, Gemmatimonadota, Verrucomicrobiota, Patescibacteria, Nitrospirota, Chytridiomycota, and Mortierellomycota were greatest near the *in situ* pH and decreased with the pH amendment. The relative abundance of Proteobacteria increased in a quadratic manner as soil pH increased, and was more sensitive to the alkaline treatment.

For the top 50 bacterial taxa in abundance at the family level, 3 and 33 taxa in relation to the amended pH conformed to the linear and quadratic fittings in Dezhou soil, respectively. The relative abundances of Comamonadaceae, S0134_terrestrial_group, and Rokubacteriales increased linearly with the increase of the amended pH (Fig. S7). The relative abundances of Vicinamibacteraceae, Bryobacteraceae, and Saprospiraceae were high near the *in situ* pH and decreased with the pH amendment, whereas the variations in Sphingomonadaceae, Xanthomonadaceae, Sphingobacteriaceae, and Saccharimonadales showed the opposite trend (Fig. S8). The relative abundance of Sedimentibacteraceae exhibited a quadratic pattern as soil pH increased, and was more sensitive to the alkaline treatment. Two and 39 among the top 50 taxa at the family level in relation to the amended pH conformed to the linear and quadratic fittings in Wuxi soil, respectively. The relative abundance of Rhodanobacteraceae had a negative linear correlation while the relative abundance of Xanthomonadaceae had a positive linear correlation with the amended pH, even if both were from the same phylum (Fig. S7). Figure S9 displays taxa with good quadratic fits to the amended pH (R^2^ > 0.80, *P* < 0.001) in Wuxi soil; the relative abundances of these taxa except Comamonadaceae, Blastocatellaceae, Intrasporangiaceae, and Clostridiaceae reached the lowest or highest peaks near the *in situ* pH.

### Response of functional genes to site and pH amendment

**Fig 7:**
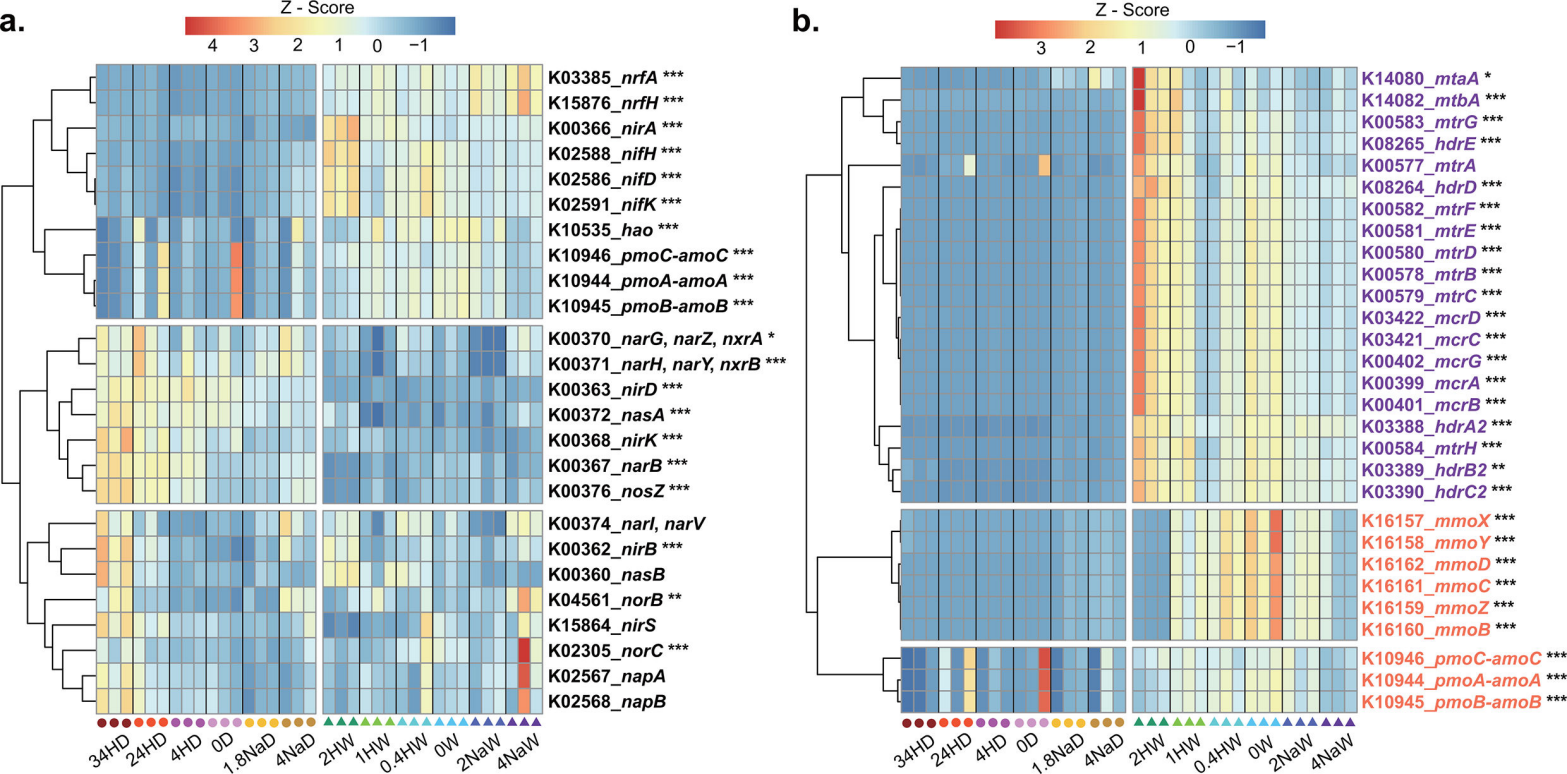
Predicted abundances of functional genes that participate in the nitrogen cycle (a), methane production and consumption (b) by PICRUSt2 across treatments and sites. The gene abundances were standardized by Z-Score. Hierarchical clustering of the genes was used to create blocks in which the values are close. Enzymes encoded by these genes (a) perform the nitrogen transformations according to KEGG (Kyoto Encyclopedia of Genes and Genomes) Orthology: assimilatory nitrate reductase (NAS, *nasAB*); membrane-bound (NAR, *narBGHIVYZ*) and periplasmic (NAP, *napAB*) dissimilatory nitrate reductases; nitrite oxidoreductase (NXR, *nxrAB*); nitrite reductases (NIR, *nirABDKS*); dissimilatory periplasmic cytochrome c nitrite reductase (ccNIR, *nrfAH*); nitric oxide reductase (NOR, *norBC*); nitrous oxide reductase (NOS, *nosZ*); molybdenum-iron nitrogenases (MoFe, *nifHDK*); ammonia monooxygenase (AMO, *amoCAB*); and hydroxylamine oxidoreductase (HAO, *hao*). The purple font indicates the genes relevant with methane production, and the orange font indicates the genes relevant with methane consumption (b). Significant differences in the two sites were based on MaAsLin 2. Asterisks denote different significance levels: *P < 0.05, **P < 0.01, and ***P < 0.001. D, Dezhou; H, H_2_SO_4_; Na, NaOH; and W, Wuxi.

The predicted abundances of functional genes involved in the nitrogen cycle, and methane production and consumption were closely relevant with experimental site and amended pH. These functional genes were clustered into three groups (Fig. 7a). For the first group, the abundances of *nrfAH*, *nirA*, *nif HDK*, *hao*, and *amoCAB* genes were significantly greater in Wuxi soil than in Dezhou soil. For the second group, significantly greater abundances of *narBGHYZ*, *nxrAB*, *nirD*, *nasA*, *nirK*, and *nosZ* were observed in Dezhou soil than in Wuxi soil. For the third group, only the abundances of *nirB* and *norBC* genes were significantly greater in Wuxi soil than in Dezhou soil. Functional genes of methane production and consumption were also clustered into three groups, which coincided with their functions (Fig. 7b). The genes relevant with methane production were clustered into one group, whereas genes encoding particulate methane monooxygenase (*pmoCAB*) and soluble methane monooxygenase (*mmoBCDXYZ*) were clustered into one group, respectively. Except for *mtrA* gene, the abundances of all other genes in Wuxi soil were significantly greater than those in Dezhou soil. Treatments received the highest acid and alkali (34HD, 4NaD, 2HW, and 4NaW) had the most functional genes, which significantly differed from those in the controls (0D or 0W) (Fig. S10a through d). In Dezhou soil, the *nosZ* gene abundance in 34HD, 24HD, 4HD, and 4NaD treatments was significantly higher than that in the 0D control treatment. In Wuxi soil, the abundances of *amoA*, *amoB*, and *hao* genes in 2HW and 4NaW treatments were significantly lower than those in the 0W control treatment. The abundances of key methanotrophic genes (*mmoBCDXYZ* and *pmoAB*) involved in the methane oxidation process were significantly lower in 2HW treatment compared to the 0W control treatment.

## DISCUSSION

### Soil pH amendment determined microbial diversity and composition

Studies have documented that soil pH has a strong influence on the diversity and composition of soil bacterial community at a regional scale (11, 21, 26, 27). For instance, the bacterial diversity decreased with increasing elevation, where pH contributes most to the diversity variation (27). Moreover, these studies investigated the relationships between the pH and bacterial communities in different soils subjected to the variations of land use (11), cross-continent (21), elevational gradient (27), and vegetation type (26). Numerous soil and site characteristics (for example, soil moisture, nutrient availability, and climate change) often co-vary with changes in soil pH, thus it is impossible to know whether pH itself is the major factor shaping soil bacterial communities. In this study, we confirmed that pH governed soil bacterial community in pH-manipulated soils. Our results corroborated that the alpha diversities of bacterial community were highest near their *in situ* pHs in soils under two contrasting cropping systems, and their strong relationships were also conformed to a quadratic fitting (Fig. S4). It is consistent with the finding that the optimal pH for bacterial community is highly relevant to *in situ* pH in various soil types (28, 49). Furthermore, a 1.7-unit change of *in situ* pH led to a 50% decrease in bacterial activity (28). Treatments that were supplemented with the highest acid or alkaline in Dezhou (34HD and 4NaD) and Wuxi (2HW and 4NaW) soils significantly reduced bacterial diversity indices compared to the controls (0D and 0W), with pH changes of more than 1.7 unit for the 34HD, 2HW, and 4NaW treatments.

In addition to the alpha diversity, our results established that soil pH is a vital driving factor of soil bacterial community composition. The bacterial community composition was most influenced by amended soil pH during the experiment of pH amendment (Fig. 3c). The variation partition analysis showed that the bacterial community composition in Dezhou soil was mainly determined by pH_A_, C/N, DOC, and NO_3_^−^-N, whereas that in Wuxi soil was mainly determined by NO_3_^−^-N, DOC, pH_A_, and C/N (Fig. 3e). The amended pH and key physicochemical properties strongly influenced the bacterial communities in these two agricultural soils. These results confirmed the hypothesis in previous studies that pH is a vital driving factor of bacterial community composition (21, 22). Our results demonstrated that soil bacterial community was shaped both directly and indirectly by pH. Above all, soil pH plays a pivotal role in membrane-bound proton pumps and protein stability (50), thus pH directly imposes a physiological constraint on soil bacteria. Subsequently, soil pH indirectly alters bacterial community composition by soil characteristics (for example, nutrient availability) that are often closely related to soil pH.

### Soil pH amendment weakly correlated with fungi compared with bacteria

Compared to the bacterial community, the influence of soil pH on the fungal community has received less attention. Our findings suggested that although the diversity and composition of fungal community were related to soil pH, the effects were considerably weaker than that of the bacterial community. Overall fungal diversity was not significantly correlated with the amended pH in these two agricultural soils (Fig. S4c and d). Individual impacts of the amended pH on fungal community composition were far less pronounced than on bacterial community composition in both agricultural soils (Fig. 3e and f). Moreover, the relative abundances of major fungal phyla exhibited considerably weaker responses to the changes in pH compared to major bacterial phyla (Fig. 6). These results partially align with our expectations, as fungi are believed to possess a greater capability to tolerate pH stress than bacteria due to their stronger cell walls and unique mycelial networks (51). Physiological studies of bacterial and fungal pure cultures suggest that fungal taxa typically exhibit a wider pH optimum, spanning 5–9 pH units without significant growth inhibition, whereas bacterial species tend to have a narrow pH range for growth in pure culture, often varying within 3–4 pH units between the maximum and minimum (22, 52, 53). It is plausible that competitive interactions between fungi and bacteria may be influencing the observed shifts in the fungal community across a steep gradient of bacterial community change (22). The observed pattern between the fungal community and soil pH could be an indirect effect, mediated by the bacterial community, which is highly dynamic along the soil pH gradient (22, 54).

### Soil pH amendment as a reliable predictor of bacterial taxa abundance

Previous studies have established a strong association between soil pH and the shifts in the relative abundance of specific taxa at the phylum level (21–23, 29). It is worth noting that we have explored the complete response curves of soil bacterial taxa to soil pH, rather than focusing on the effect of acidification like many previous studies (26, 55). The relative abundances of many dominant bacterial phyla were significantly related to the amended soil pH that was regulated by added acid or alkaline, and the relationships between most of them and the pH conformed to a quadratic fitting (Fig. 6). It is consistent with the finding that the pH dependency of bacterial growth is modeled using a simple second-degree function in most cases (28).

The relative abundances of some dominant bacterial phyla in response to the pH gradient differed in Dezhou and Wuxi soils, which were also inconsistent with other studies (22, 23, 29). The reason might be that the relative abundances of these dominant bacterial phyla were closely relevant to the variations of site characteristics (e.g., vegetation type, texture, and climate). For instance, the relative abundance of Proteobacteria in Dezhou soil was least near the *in situ* pH and increased with pH amendment, whereas that in Wuxi soil increased in a quadratic manner as soil pH increased. Our results are unlike the finding of a linear positive correlation between the relative abundance of Proteobacteria and soil pH as reported previously (22, 23). Moreover, the high abundance of Proteobacteria was associated with an increased carbon availability (56). The relative abundance of Proteobacteria was increased in treatments under high DOC in Wuxi soil but not in Dezhou soil (Fig. 6; Fig. S3).

The relative abundance of Bacteroidota was decreased to a minimum as soil pH was decreased in Wuxi (Fig. 6), possibly because most bacteria belonging to Bacteroidota are Gram-negative bacteria with relatively thin cell walls (56). Conversely, the relative abundance of Actinobacteriota was increased as soil pH was decreased in both Dezhou and Wuxi (Fig. 5 and 6), perhaps due to their Gram-positive cell walls, which confer more resistance to acid stress (57). Similarly, Firmicutes, also Gram-positive bacteria (56), exhibited their highest relative abundances when soil pH reached either its minimum in Wuxi or its maximum in Dezhou (Fig. 5 and 6).

We also explored the relationships between soil pH and the relative abundances of dominant taxa at the family level, which provided a finer taxonomic resolution (Fig. S7 to S9). We selected the top 50 bacterial taxa in abundance at the family level, and more than 70% of them in relation to the amended pH conformed to a linear or quadratic fitting in both soils. The taxa at the family level responded differently to soil pH even if they belong to the same phylum. For instance, Comamonadaceae, Sphingomonadaceae, and Xanthomonadaceae in Dezhou, and Rhodanobacteraceae, Xanthomonadaceae, and Comamonadaceae in Wuxi belong to the Proteobacteria, but their relative abundances varied to soil pH in diverse manners on a single soil type (Fig. S9). Because not all bacterial taxa within the same group shifted in a similar manner, it appeared to be more reasonable to evaluate the response of bacterial community to soil pH at a lower taxonomic level (58). Overall, the pH beyond a certain range has resulted in a direct effect on bacterial growth and interaction, which in turn affects diversity, composition, and functionality of soil bacterial community (7, 50, 59). A difference in pH units indicates a 10-fold change in hydrogen ion concentration, which significantly impacts the acidity of soils. Even small changes in pH can lead to substantial shifts in soil chemistry and subsequent biological processes. Although our study primarily focused on the pH levels and their immediate effects on the relative abundances of dominant taxa at the phylum and family levels, it is important for future research to consider how changes in pH might influence the redox potential (mV) and subsequent metabolism of single cell organisms, particularly in soils with limited buffering capacity or those extensively modified by human activities.

### Soil pH amendment affected potential nutrient cycling

It is well established that soil microorganisms have crucial roles in nutrient cycling and in the maintenance of ecosystem functions in terrestrial ecosystems (2–4). Given that soil pH has a significant influence on the diversity and composition of soil bacterial community, it is crucial to investigate the effect of soil pH amendment on nutrient cycling. In this study, we chose Dezhou and Wuxi soils, which are the hotspots of greenhouse gas emissions (CH_4_ and N_2_O) from agricultural sources (30, 33). We focused on the transformation of nitrogen and the abundances of functional genes involved in the nitrogen cycle, as well as in methane production and consumption in relation to soil pH amendment. We found that 34HD and 24HD treatments accelerated the conversion rates of nitrogen (consumption rate of NH_4_^+^-N and production rate of NO_3_^−^-N) in the Dezhou soil, whereas 2HW and 1HW were opposite in the Wuxi soil (Fig. S2 and S3). Because the optimum pH for urease activity ranges from pH 5 to 8 (60), the decomposition of urea under 2HY treatment was enormously hindered. The treatments that received the highest acid and alkali had the greatest number of functional genes that were significantly different from the controls (Fig. S10). These functional genes were involved in the syntheses of key enzymes for nitrogen fixation, nitrification, denitrification, methane production and consumption. It is consistent with the effects of soil pH on the nitrogen cycle, and methane production, and consumption (61–63). For example, soil pH significantly affected the abundance of the nosZ gene, which can encode N_2_O reductase. Except for the 1.8NaD treatment, other acid or alkali treatments significantly increased the abundance of the nosZ gene compared to the 0D control treatment in Dezhou soil (Fig. S10a). Moreover, the abundances of most key methanotrophic genes involved in the methane oxidation process were affected by soil pH amendment (Fig. S10c and d). Future work is necessary to combine greenhouse gas N_2_O and CH_4_ emission data to further explore the microbiological mechanisms by which soil pH affects greenhouse gas emissions.

In addition to the effect of pH, variations in site characteristics such as vegetation type, texture, and climate also significantly impacted the abundances of functional genes involved in carbon and nitrogen cycles (Fig. 7). For instance, the abundances of almost all functional genes involving methane production and consumption were notably lower in Dezhou soils than in Wuxi soils, which were likely attributed to the year-round cultivation of rice in Wuxi soils. The anaerobic habitats in flooded rice fields serve as significant sources of methane (64). Due to the limited accuracy of functional prediction, future studies should use real-time quantitative polymerase chain reaction and metagenomics to further understand the effect of soil pH amendment on nutrient cycling and ecosystem functions.

### Conclusions

The diversity and composition of bacterial and fungal communities were associated with changes in soil pH in two agricultural soils, but the influence on fungal community was weaker than on the bacterial community. As expected, soil pH had both direct and indirect effects on bacterial diversity, composition, and taxa relative abundances. Alpha diversity of the bacterial community was highest near the *in situ* pH in both soils, and their strong relationships conformed to a quadratic fitting. The relative abundances of diverse dominant bacterial taxa at the phylum and family levels were both related to the amended soil pH, and the relationships between most of these taxa and pH conformed to a quadratic fitting. Moreover, soil pH amendment could affect the transformation of nitrogen and the predicted abundances of functional genes involved in the nitrogen cycle, and methane production and consumption. Our findings are pivotal for developing more effective soil management and conservation strategies.

## SUPPLEMENTAL MATERIAL

Supplemental material spectrum.04165-23-s0001.docx Table S1; Fig. S1 to S10.

ASM does not own the copyrights to Supplemental Material that may be linked to, or accessed through, an article. The authors have granted ASM a non-exclusive, world-wide license to publish the Supplemental Material files. Please contact the corresponding author directly for reuse.
