# Supplementary material for: Soil pH amendment alters the abundance, diversity, and composition of microbial communities in two contrasting agricultural soils
Source: Microbiol Spectr. 2024 Jun 25;12(8):e04165-23. doi: 10.1128/spectrum.04165-23 (PMC11302230; doi:10.1128/spectrum.04165-23)
Supplement: Supplemental material — Table S1; Fig. S1 to S10. [file spectrum.04165-23-s0001.docx]

# Supplementary Material

# Supplementary table

Table S1 Basic physicochemical properties of two contrasting soils in Dezhou (Ochric Aquic Cambosols) and Wuxi (Stagnic Anthrosols)

| Site | pH  (H_2_O) | Total C  (%) | Total N  (%) | NH_4_^+^-N (mg kg^−1^) | NO_3_^−^-N (mg kg^−1^) | DOC  (mg kg^−1^) | Water content  (%) | Water holding capacity  (%) |
| --- | --- | --- | --- | --- | --- | --- | --- | --- |
| Dezhou | 8.43 a | 1.94 a | 0.13 b | 0.57 a | 111.5 b | 308.1 a | 7.10 a | 39.25 b |
| Wuxi | 6.17 b | 1.22 b | 0.14 a | 0.96 a | 162.2 a | 266.0 a | 13.62 a | 61.45 a |

The same letter indicates insignificant difference between sites at *P* < 0.05 as determined by *t*-test.

# Supplementary figures

Figure S1. Rarefaction curves showing saturation of sequencing. Rarefaction curves of bacterial 16S rRNA gene or fungal ITS gene are labeled with (a and c) site or (b and d) pH treatment. Abbreviations: D, Dezhou; H, H_2_SO_4_; Na, NaOH; and W, Wuxi; same as follows. See detailed treatment information in Table 1.

Figure S2. Changes in soil physicochemical properties under different treatments in Dezhou soils (*n* = 3; error bars represent standard errors). The pH value was the negative logarithm transformation of the hydronium ion concentration. The details of treatments are in Table 1.

Figure S3. Changes in soil physicochemical properties under different treatments in Wuxi soils (*n* = 3; error bars represent standard errors). The pH value was the negative logarithm transformation of the hydronium ion concentration. The details of treatments are in Table 1.

Figure S4. Shannon indices of bacterial (a and b) and fungal (c and d) communities in relation to soil initial pH in Dezhou and Wuxi soils. Lines represent the best-fit quadratic model to the data. The coefficients of determination (R^2^) are shown in yellow and blue for Dezhou and Wuxi, respectively. Shadow represents a 95% confidence level. The pH value was the negative logarithm transformation of the hydronium ion concentration. Asterisks denote different significance levels: * *P* < 0.05, ** *P* < 0.01, and *** *P* < 0.001.

Figure S5. Volcano plot of results from differential abundance analysis using ANCOM across treatments in Dezhou (a) and Wuxi (b) soils, and between sites (c) for the dominant bacterial phyla. The W statistic represents the strength of the test and is the number of times the null-hypothesis was rejected for a given species. Taxa above the dashed line are statistically significant. The x-axis value presents the effect size as the clr (centered log ratio) by f-statistic among treatments, or by transformed mean difference between the two sites. For the third panel, a positive x-axis value means the phylum is abundant in Dezhou compared to Wuxi or vice versa for a negative x-axis value.

Figure S6. Volcano plot of results from differential abundance analysis using ANCOM across treatments in Dezhou (a) and Wuxi (b) soils, and between sites (c) for the dominant fungal phyla. The W statistic represents the strength of the test and is the number of times the null-hypothesis was rejected for a given species. Taxa above the dashed line are statistically significant. The x-axis value presents the effect size as the clr (centered log ratio) by f-statistic among treatments, or by transformed mean difference between the two sites. For the third panel, a positive x-axis value means the phylum is abundant in Wuxi compared to Dezhou or vice versa for a negative x-axis value.

Figure S7. Correlations between the relative abundances of dominant bacterial taxa (top 50 in abundance) at the family level and soil initial pH. Lines represent the best-fit linear models to the data. Pearson correlations coefficients (R) are shown for each taxon with *P*-values in yellow and blue for Dezhou and Wuxi, respectively. p indicates the phylum to which the taxon belongs. Shadow represents a 95% confidence level. The pH value was the negative logarithm transformation of the hydronium ion concentration. Asterisks denote different significance levels: * *P* < 0.05, ** *P* < 0.01, and *** *P* < 0.001.

Figure S8. Relative abundances of dominant bacterial taxa (top 50 in abundance and R^2^ > 0.80) at the family level in relation to soil initial pH in Dezhou. Lines represent the best-fit quadratic models to the data. The coefficients of determination (R^2^) are shown for each taxon with *P*-values. p indicates the phylum to which the taxon belongs to. Shadow represents a 95% confidence level. The pH value was the negative logarithm transformation of the hydronium ion concentration. Asterisks denote different significance levels: * *P* < 0.05, ** *P* < 0.01, and *** *P* < 0.001.

Figure S9. Relative abundances of dominant bacterial taxa (top 50 in abundance and R^2^ > 0.80) at the family level in relation to soil initial pH in Wuxi. Lines represent the best-fit quadratic models to the data. The coefficients of determination (R^2^) are shown for each taxon with *P*-values. p indicates the phylum to which the taxon belongs to. Shadow represents a 95% confidence level. Asterisks denote different significance levels: * *P* < 0.05, ** *P* < 0.01, and *** *P* < 0.001.

Figure S10. Differential abundance analysis of functional genes across treatments using MaAsLin 2. Genes of the nitrogen cycle that were significantly different from controls (0D or 0W) are shown in Dezhou (a) and Wuxi (b) soils. Genes of methane production and consumption that were significantly different from the controls (0D or 0W) are shown in Dezhou (c) and Wuxi (d) soils. The absolute value of significant association indicates the magnitude of the difference. Plus and minus signs indicate abundance above or below control, respectively.

## Figure S1


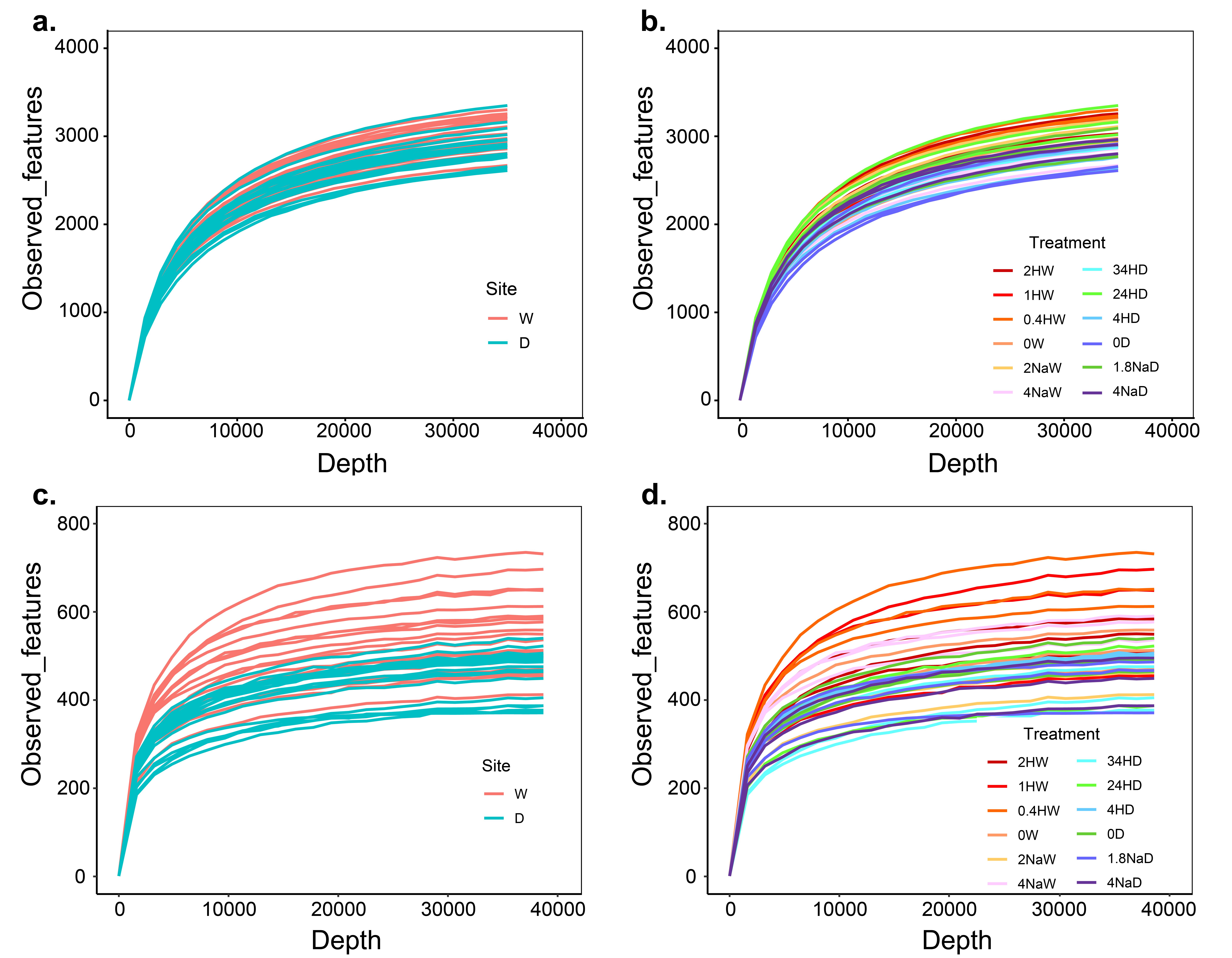


## Figure S2





## Figure S3





## Figure S4


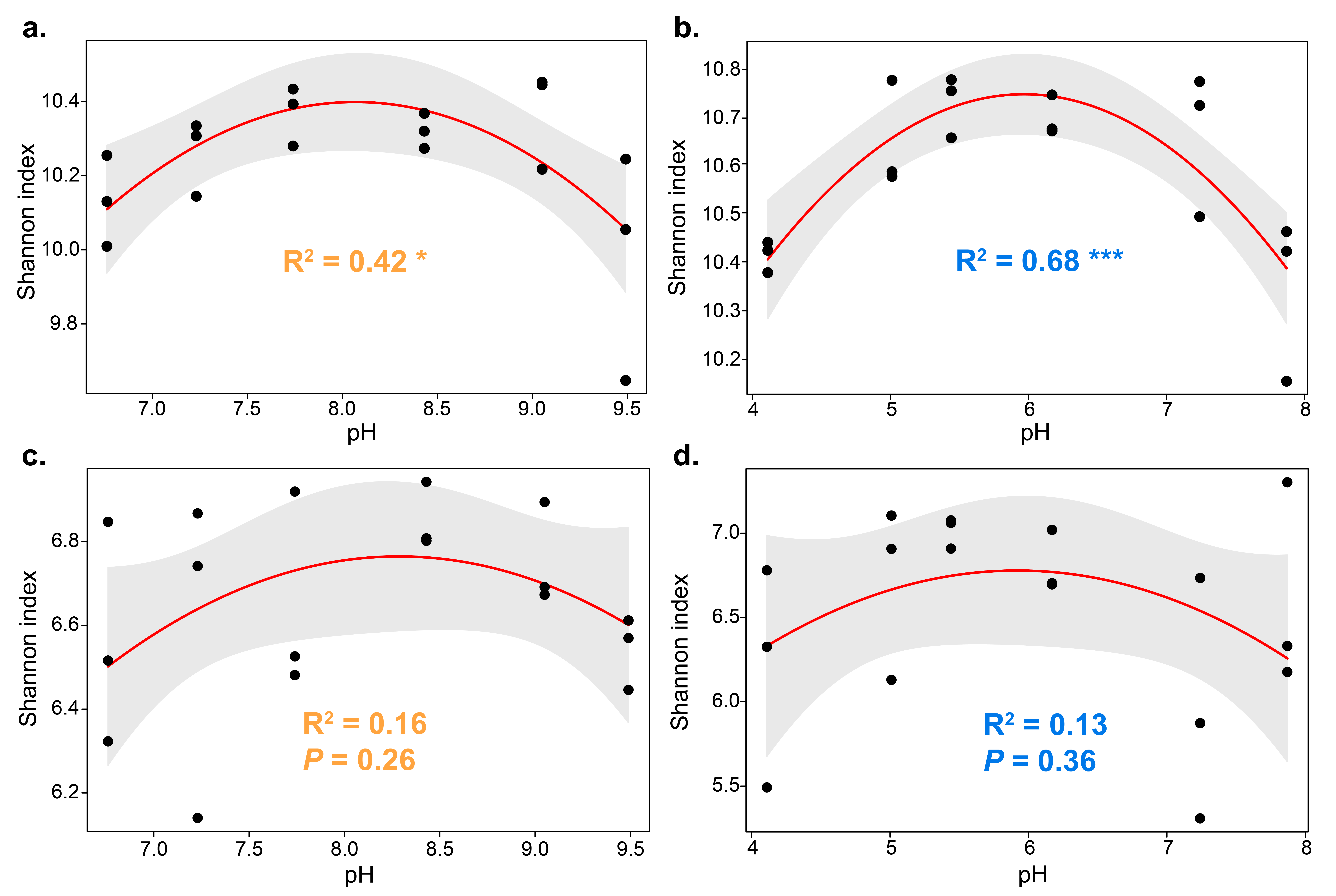


## Figure S5


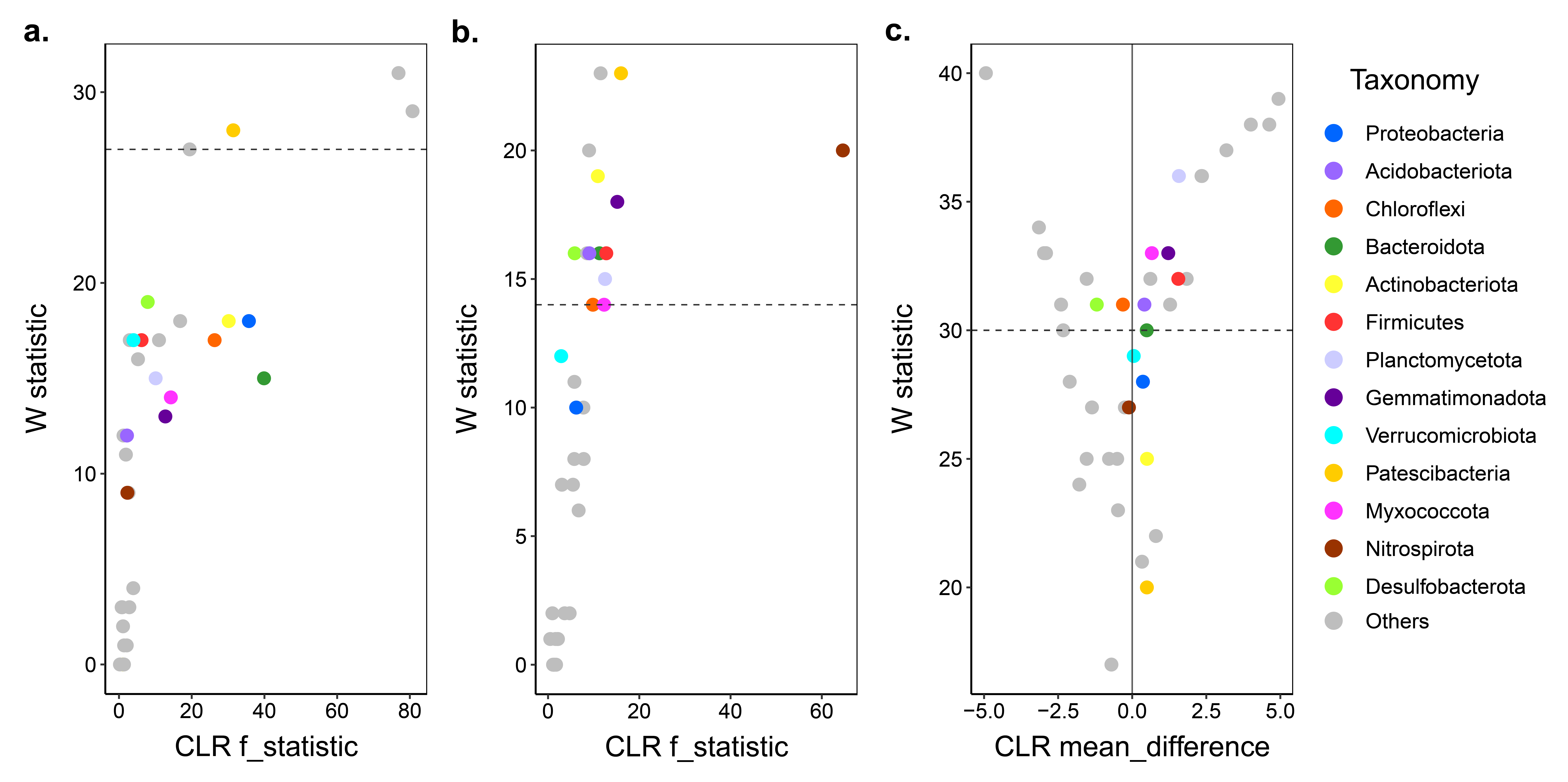


## Figure S6


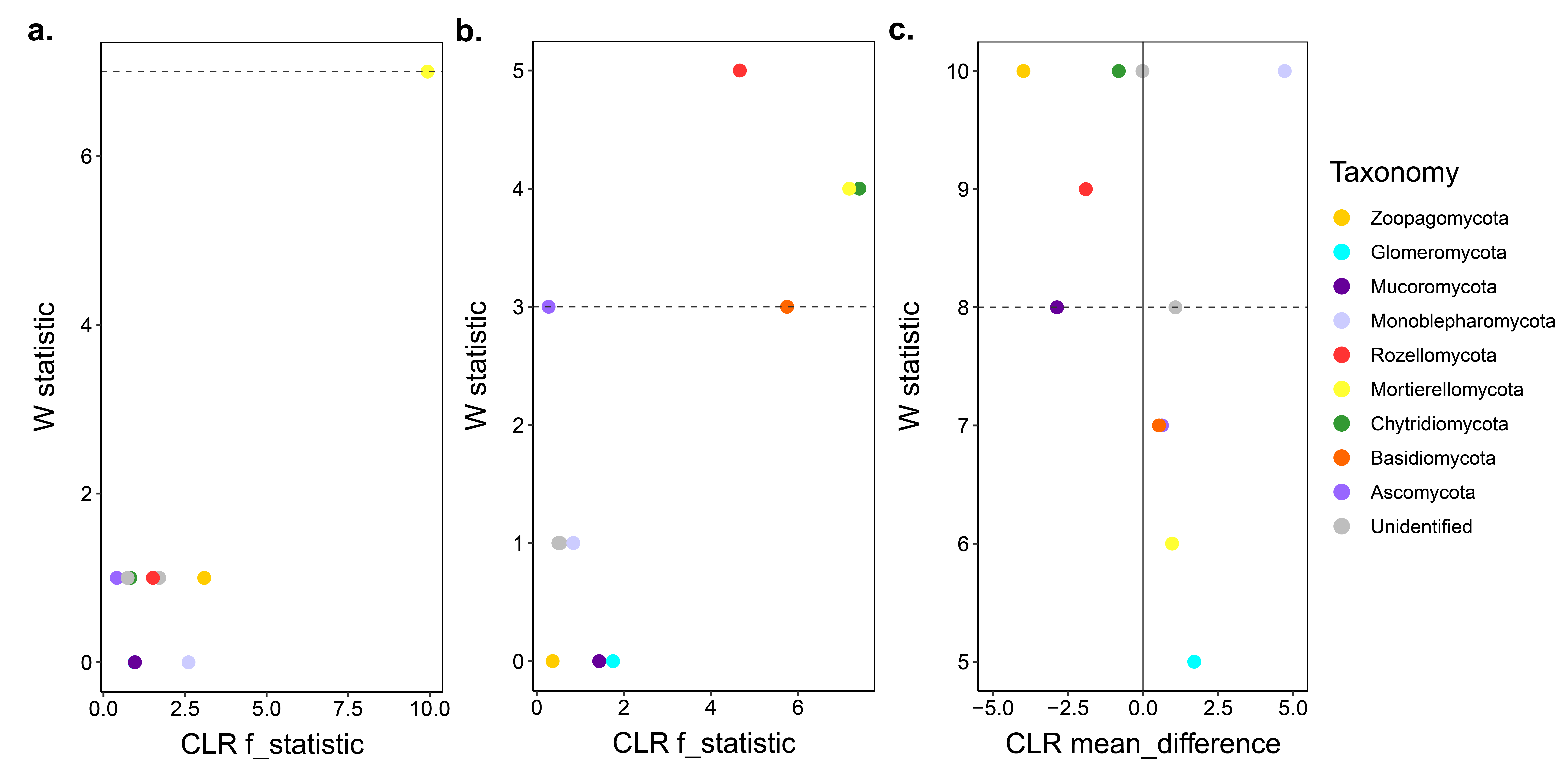


## Figure S7





## Figure S8





## Figure S9





## Figure S10
